# Identification of Volatile Organic Compounds Emitted by Two Beneficial Endophytic *Pseudomonas* Strains from Olive Roots

**DOI:** 10.3390/plants11030318

**Published:** 2022-01-25

**Authors:** Nuria Montes-Osuna, Tomislav Cernava, Carmen Gómez-Lama Cabanás, Gabriele Berg, Jesús Mercado-Blanco

**Affiliations:** 1 Departamento de Protección de Cultivos, Instituto de Agricultura Sostenible, Agencia Estatal Consejo Superior de Investigaciones Científicas (CSIC), Avenida Menéndez Pidal s/n, Campus “Alameda del Obispo”, 14004 Cordoba, Spain; nuriamontes@ias.csic.es (N.M.-O.); cgomezlama@ias.csic.es (C.G.-L.C.); 2 Institute of Environmental Biotechnology, Graz University of Technology, Petersgasse 12/I, 8010 Graz, Austria; tomislav.cernava@tugraz.at (T.C.); gabriele.berg@tugraz.at (G.B.); 3 Leibniz-Institute for Agricultural Engineering Potsdam, Max-Eyth-Allee 100, 14469 Potsdam, Germany; 4 Institute for Biochemistry and Biology, University of Potsdam, Karl-Liebknecht-Str. 24/25, 14476 Potsdam, Germany

**Keywords:** biological control agents, olive rhizobacteria, *Pseudomonas* sp. PICF6, *Pseudomonas simiae* PICF7, root endophytes, *Verticillium dahliae*, volatilome

## Abstract

The production of volatile organic compounds (VOCs) represents a promising strategy of plant-beneficial bacteria to control soil-borne phytopathogens. *Pseudomonas* sp. PICF6 and *Pseudomonas simiae* PICF7 are two indigenous inhabitants of olive roots displaying effective biological control against *Verticillium dahliae.* Additionally, strain PICF7 is able to promote the growth of barley and *Arabidopsis thaliana*, VOCs being involved in the growth of the latter species. In this study, the antagonistic capacity of these endophytic bacteria against relevant phytopathogens (*Verticillium* spp., *Rhizoctonia solani*, *Sclerotinia sclerotiorum* and *Fusarium oxysporum* f.sp. *lycopersici*) was assessed. Under in vitro conditions, PICF6 and PICF7 were only able to antagonize representative isolates of *V. dahliae* and *V. longisporum*. Remarkably, both strains produced an impressive portfolio of up to twenty VOCs, that included compounds with reported antifungal (e.g., 1-undecene, (methyldisulfanyl) methane and 1-decene) or plant growth promoting (e.g., tridecane, 1-decene) activities. Moreover, their volatilomes differed strongly in the absence and presence of *V. dahliae*. For example, when co incubated with the defoliating pathotype of *V. dahliae,* the antifungal compound 4-methyl-2,6-bis(2-methyl-2-propanyl)phenol was produced. Results suggest that volatiles emitted by these endophytes may differ in their modes of action, and that potential benefits for the host needs further investigation *in planta*.

## 1. Introduction

Soil-borne phytopathogens constitute a major threat affecting crops around the world, compromising global food production and security. Some of them can survive in soils for many years in the absence of host plants due to different resistance structures (i.e., microsclerotia, sclerotia, chlamydospores or oospores), hindering their effective control [1]. Examples of important soil-borne fungi causing serious yield losses in a broad crop range are some species of the genus *Verticillium* [2,3], *Fusarium oxysporum* (different *formae speciales*) [4,5], *Rhizoctonia solani* Kühn [6,7] or *Sclerotinia sclerotiorum* (Lib.) de Bary [8]. For instance, to name an example of particular interest in our study, Verticillium wilt (*Verticillium dahliae* Kleb.) is considered one of the most threatening biotic constrains for olive (*Olea europaea* L.) cultivation and the main limiting factor for olive oil production [9]. These phytopathogenic fungi were traditionally managed by crop rotations and soil treatments. Today, under intense conditions, management is usually performed by conventional chemical methods which imply the application of broad-spectrum fungicides. Nevertheless, the lack of specificity, their negative impacts on soil microbiota and/or the possibility to generate resistance are, among others, undesirable side effects in crop protection [10,11]. Thus, more environmentally friendly alternatives to control plant diseases are gaining attraction. Among them, biological control represents an interesting option within integrated management strategies.

Phytobiome studies have revealed that many plant-associated microorganisms (especially fungi and bacteria) contribute to protect plants against biotic and abiotic stresses [12,13,14,15,16]. Among these microorganisms, those that are able to develop an endophytic lifestyle without causing deleterious effects in their hosts [17,18] pose interesting perspectives from the agro-biotechnological point of view [19,20]. For example, olive roots constitute an important reservoir of beneficial endophytic and epiphytic microorganisms [21,22,23]. Some of them have demonstrated their effectiveness as biological control agents (BCAs) or plant growth promoters (PGPs), thereby constituting an eco-friendly alternative to the traditional chemically-based plant disease control and intensive crop fertilization approaches [24,25,26,27]. A collection of bacteria originating from the rhizosphere/roots of nursery-produced olive plants was generated in the frame of previous studies [28,29]. Using a holistic strategy based on in vitro antagonism tests, phenotypic and metabolic characterization, in silico identification of genetic factors involved in plant-bacteria interactions and *in planta* bioassays, the ability of some of these rhizobacteria to counteract *V. dahliae* in olive plants has been demonstrated [28,29,30,31,32,33,34,35]. Among them, *Pseudomonas simiae* PICF7 [33,35] stands out as a well-known and versatile BCA and/or PGP, not only in its natural host (olive) but also in distant plant species such as *Arabidopsis thaliana*, barley (*Hordeum vulgare* L.) and banana (*Musa acuminata* L. AAA group, cv. Cavendish) [27,36,37,38]. In contrast, the available information for another beneficial olive-derived rhizobacterium, *Pseudomonas* sp. PICF6, is still very limited except for its effectiveness to antagonize *V. dahliae* in vitro and to control Verticillium wilt of olive (VWO) [28]. Interestingly, both PICF6 and PICF7 are able to colonize the interior of olive roots [39,40].

Biological control is a multifaceted process in which competition for nutrients and niches, synthesis of extracellular enzymes or production of inhibitory compounds like antibiotics or volatile organic compounds (VOCs) can be involved [41]. In specific cases, the prevailing interference of beneficial microorganisms with phytopathogens can be based on bioactive VOCs [42,43,44,45,46]. VOCs are small organic molecules with a high vapor pressure which have been recognized as key players in the control of several plant pathogens [16,47,48]. The diversity of VOCs produced by PGPs microorganisms is high and some of these molecules are unique to particular bacterial or fungal species [49]. Numerous examples of the inhibitory effects of VOCs produced by different *Pseudomonas* spp. are available in the literature. For instance, *Pseudomonas chlororaphis* subsp. *aureofaciens* SPS-41 inhibited the mycelial growth and spore germination of *Ceratocystis fimbriata* through VOCs production [50]. Similarly, the inhibitory potential of *Pseudomonas fluorescens* ZX against *Penicillium italicum* was assayed in different media showing that diverse VOCs produced by this BCA hindered the mycelial growth and conidial germination of *P. italicum* [51]. Likewise, VOCs produced by three different isolates of *P.* *fluorescens* (strains 1–112, 2–28 and 4–6) completely inhibited the spore germination of the fungal pathogen *Penicillium expansum* [52]. In addition to antimicrobial activity, there is increasing interest in the understanding of volatile signaling in the plant-associated microbiota [14].

So far, no information is available on volatiles emitted by *Pseudomonas* sp. PICF6 and *P. simiae* PICF7, nor whether these compounds could be involved in the control of VWO. Consequently, the objectives of this study were: (i) to first assess whether strains PICF6 and PICF7 antagonize selected plant pathogens causing important losses in relevant crops, (ii) to elucidate the volatilomes of these two olive root endophytes in the absence/presence of *V. dahliae*, and (iii) to determine whether VOCs emitted by these strains are involved in the in vitro antagonism against the olive defoliating pathotype of *V. dahliae*.

## 2. Results

### 2.1. Assessment of In Vitro Antagonism against Selected Fungal Phytopathogens

Results from in vitro antagonism tests showed that *Pseudomonas* sp. PICF6 and *P. simiae* PICF7 only inhibited the growth of *Verticillium longisporum* ELV25 and *V. dahliae* V937I (Table 1 and Supplementary Figure S1). In contrast, *Paenibacillus polymyxa* PIC73, another beneficial olive rhizobacteria used in this study for comparative purposes due to its broad antagonist activity [24], effectively inhibited *S. sclerotiorum*, *R. solani* and *V. longisporum* ELV25 in both assayed media (Table 1 and Supplementary Figure S1). The only exception was *F. oxysporum* f. sp. *lycopersici Fol* 007 that was not inhibited in NA medium (Table 1). 

### 2.2. Elucidation of the Volatilomes of Strains PICF6 and PICF7

Analysis by Headspace Solid Phase Microextraction (HS-SPME) Gas Chromatography-Mass Spectrometry (GC-MS) of VOCs profiles of *Pseudomonas* sp. PICF6 and *P. simiae* PICF7 cultivated alone or in the presence of *V. dahliae* V937I, as well as the VOCs profile of this representative of the defoliating (highly-virulent) pathotype causing severe VWO, indicated that both the endohytic bacteria and the pathogen are able to produce diverse compounds (Table 2 and Supplementary Figure S2). 

On the one hand, four VOCs, namely 2-methyl-1-propanol, 3-methyl-1-butanol, 2-methyl-1-butanol and 2-phenylethanol were exclusively detected in the VOC profile of *V. dahliae* V937I. On the other hand, several compounds were emitted by the endophytic BCAs here studied and their production varied depending on the experimental conditions (i.e., presence/absence of *V. dahliae*). Indeed, fourteen compounds were detected when *Pseudomonas* sp. PICF6 was cultivated alone. However, in the presence of the pathogen, 20 compounds were detected for this olive endophyte, 13 of them being produced in both conditions (Table 2). Some of them (e.g., dimethyltrisulfane, tridecane, 1-decene, 1,10-undecadiene, etc.) have been earlier described in the literature for their antimicrobial activity or for their involvement in plant growth promotion (Table 2). For *P. simiae* PICF7, 15 different VOCs were identified. Twelve of these compounds were also produced when this BCA was incubated in the presence of *V. dahliae* V937I. However, other compounds such as bis(methylsulfanyl)methane, 4-methyl-2-pentanone and 2-decyloxiranese (produced by PICF7 but not by PICF6) were identified only when the bacterium was incubated alone, while 4-methyl-2,6-bis(2-methyl-2-propanyl)phenol was identified when PICF7 was incubated in the presence of isolate V937I (Table 2).

Results showed that strains PICF6 and PICF7 shared 12 VOCs when they were incubated alone. However, in the presence of *V. dahliae* V937I, only 11 VOCs were emitted. 4-methyl-2-pentanone was produced by PICF7 but not by PICF6, regardless of whether or not the latter BCA was incubated alone or in the presence of *V. dahliae* V937I. In addition, bis(methylsulfanyl)methane and 2-decyloxirane were produced by PICF6, but only in the absence of the pathogen. 2,6,11-trimethyldodecane and methyl thiocyanate were exclusively produced by strain PICF6, independently of the absence or presence of the pathogen. 1-tridecyne, 2-undecanone (antifungal activity), 2-undecanol (antifungal and nematicidal activity), 2-nonanol (nematicidal activity) and 10-methyl-1-undecene were only identified when strain PICF6 was incubated with *V. dahliae* V937I (Table 2). Interestingly, tridecane (a compound related to plant growth promotion), (3Z)-3-dodecene and 2,5-dimethylpyrazine (antifungal activity) were produced by PICF6 and PICF7 only when they were incubated alone. However, these compounds were only detected for strain PICF6 when incubated with the pathogen. Finally, 4-methyl-2,6-bis (2-methyl-2-propanyl)phenol (antifungal activity) and 3,7-dimethyl-1-octene were only detected when each BCA was grown in the presence of V937I (Table 2).

### 2.3. Evaluation of the Antagonistic Effect of VOCs Emitted by Pseudomonas sp. PICF6 and Pseudomonas simiae PICF7 against Verticillium dahliae 

The Two Clamp VOCs Assay (TCVA) performed revealed that bacterial volatiles were not involved in the observed in vitro antagonistic effect of strains PICF6 and PICF7 against the defoliating pathotype of *V. dahliae*. Indeed, *V. dahliae* colonies confronted with these root endophytes showed a similar size to that scored for control plates. Statistical analysis showed no significant differences among treatments, either on potato dextrose agar (PDA) or nutrient agar (NA) culturing media (Table 3). 

## 3. Discussion

A relevant outcome of this study was to demonstrate that the two selected endophytic *Pseudomonas* strains from olive roots can produce an impressive portfolio of VOCs including those with antifungal, nematicidal and plant growth promoting effects. In addition, under in vitro experimental conditions, PICF6 and PICF7 were only able to inhibit the growth of *V. dahliae* V937I and *V. longisporum* ELV25 to some extent. Nevertheless, their effectiveness as true BCA against *V. longisporum* will need further *in planta* confirmation, in contrast to the abundant information available regarding biocontrol of VWO exerted by these strains [28,30,32,35]. This result suggested that in vitro antagonism of both olive root endophytes was specific and effective only against representatives of *Verticillium* spp. 

Volatile compounds released by beneficial rhizobacteria have been identified as a mechanism to antagonize soil-borne pathogens without physical contact between the BCA and its target [42,48]. Additionally, some VOCs produced by microorganisms are also able to stimulate the plant’s growth and to induce systemic disease resistance ([65], and references therein). In the present study, we characterized for the first time the volatilomes of the olive root endophytes PICF6 and PICF7. Many of the VOCs produced by these strains have been earlier described either for their antifungal activity or the capacity to promote plant growth. The use of these natural substances produced at large scale could be a promising alternative to the traditional use of synthetic fungicides, thereby contributing to more sustainable strategies for the control of phytopathogens [48]. For instance, direct application of some of these compounds (e.g., S-methyl ethanethioate, (methyldisulfanyl)methane, dimethyltrisulfane and 1-undecene), which are produced by PICF6 and PICF7, was earlier proven to be effective against *Alternaria alternata*, *Botrytis cinerea*, *Cochliobolus heterostrophus*, *Phytophthora infestans*, *Ralstonia solanacearum* or *R. solani* [47,54,55,60,66,67]. Moreover, (methyldisulfanyl)methane (also known as dimethyl disulfide) elicited a protective response in tobacco and corn plants against *B. cinerea* and *C. heterostrophus* under greenhouse conditions [55], and was able to promote growth of *A. thaliana* [47]. Remarkably, 4-methyl-2,6-bis(2-methyl-2-propanyl) phenol (also known as butylated hydroxytoluene) was detected in the olive rhizobacteria tested here, but only when they were co-incubated with *V. dahliae*. Recently, it has been shown that this compound, also produced by *P. polymyxa* CF05, presented a strong inhibitory effect against the pathogenic fungus *Rhizopus stolonifera* [61]. It is worth mentioning that the emission of different compounds when a BCA and a pathogen are co-incubated may be related to species-specific responses when both microorganisms share the same (micro)habitat [68]. The implications of these pathogen-induced alterations on the olive rhizobacteria volatilomes deserve further in-depth analysis.

Concerning VOCs that were shown to be specifically produced by strain PICF6, four compounds (2-undecanone, methyl thiocyanate, 2-undecanol and 2-nonanol) were detected in the presence of *V. dahliae*. Among them, 2-undecanone might be of interest because of its antifungal activity. Indeed, the volatilomes of different strains of *Burkholderia ambifaria* showed the presence of 2-undecanone. In vitro experiments demonstrated that high concentrations of this compound affect the growth of *A. alternata* and *R. solani* when pure 2-undecanone was used [47]. Similarly, methyl thiocyanate produced by *Pseudomonas donghuensis* has been reported to be involved, among other VOCs, in the strong antimicrobial activity exerted by this bacterium against the fungal pathogens *Fusarium culmorum* PV, *R. solani* and *V. dahliae* JR, and the oomycete *Pythium ultimum* P17 [54]. Mycelial growth of *V. dahliae* and *F. oxysporum* was inhibited by 2-undecanol produced by *P. polymyxa* KM2501-1 or *Bacillus velezensis* CT32 which is also one of the most active compounds, along with 2-nonanol, against the pathogenic nematode *Meloidogyne incognita* [63,64].

Several VOCs (i.e., tridecane, 2,5-dimethylpyrazine, 1-decene or 2-decyloxirane) produced by *P. simiae* PICF7 have been earlier reported to display antimicrobial activity against different phytopathogens (e.g., *Pseudomonas syringae* pv. maculicola ES4326, *Penicillium italicum*, *Sclerotinia minor*, *Pythium ultimum*, *R. solani* or *B. cinerea*) [51,57,58]. Despite the fact that different compounds that are potentially implicated in plant pathogen control were identified in the volatilomes of strains PICF6 and PICF7, the involvement of VOCs produced by these root endophytes in the antagonism towards *V. dahliae* could not be proven by the implemented Two Clamp VOCs Assay (Table 3). We thus conclude that growth inhibition of *Verticillium* spp. must be a consequence of bacterial metabolites that diffuse through the medium (Table 1 and Supplementary Figure S1), instead of bioactive VOCs. However, some relevant aspects need to be considered that may influence/alter VOCs emission by the endophytes tested here. For example, Zhang et al. [50] showed that the antifungal action of volatiles emitted by *P. chlororaphis* subsp. *aureofaciens* SPS-41 against *Ceratocystis fimbriata* was strongly influenced by the initial bacteria concentration. These authors evaluated the effect of different inoculation strategies and concentrations of strain SPS-41 on the antifungal activity of the VOCs towards the pathogen *C. fimbriata* which causes black rot disease in sweet potato tuber roots. Their study revealed that the antifungal activity of the VOCs was augmented after the inoculum concentration and the inoculation volume of strain SPS-41 were increased [50]. Therefore, we cannot completely rule out that the observed lack of inhibition towards *V. dahliae* by PICF6 and PICF7 volatilomes could be due to insufficient bacterial biomass needed to produce inhibitory amounts of a specific VOCs. It would thus be interesting to test some of these VOCs individually and at a higher concentration against *V. dahliae*. Besides, different studies have shown that the production of certain VOCs is highly dependent on the culturing medium, thereby resulting in the characterization of an unique volatilome produced under specific growing conditions [69,70]. This may have important consequences for their activity in terms of pathogen inhibition and/or plant growth promotion. For instance, significant changes in VOCs-mediated pathogen inhibition by *Lysobacter* spp. due to the culturing media used have been reported. VOCs emitted by *Lysobacter antibioticus*, *L. capsici*, *L. enzymogenes* and *L. gummosus* grown on NA inhibited the mycelial growth of *Phytophthora infestans*. Conversely, when PDA was used, the VOCs produced by these *Lysobacter* spp. did not affect the growth of the oomycete [71]. Therefore, it would be interesting to explore whether the lack of inhibitory effect of PICF6 and PICF7 against *V. dahliae* through VOCs can be overcome when the olive rhizobacteria are grown in culturing media other than the ones utilized in our study.

Regarding the potential involvement of VOCs produced by PICF6 and PICF7 in plant growth promotion, 1-decene, a compound elsewhere reported to increase the fresh weight of *A. thaliana* [59], was found in the volatilomes of both strains (Table 2). Interestingly, Desrut et al. [36] have recently reported the ability to promote the growth of *A. thaliana* seedlings by volatiles (not determined) emitted by strain PICF7. As mentioned above for the case of pathogen inhibition, attention should be called here since results may also vary depending on the culturing medium used. In the latter study, Murashige and Skoog (MS) medium was used. However, Blom et al. [69] did not observe growth promotion of *A. thaliana* by *P. chlororaphis* or *Pseudomonas putida* ISOf through the action of volatiles when MS medium was employed. In contrast, plant growth was enhanced when Luria–Bertani (LB) and Methyl Red Vogues Proskauer (MR-VP) were used, media that favored the production of the volatile butanediol. Whether 1-decene is produced by strain PICF7 in MS medium (as here reported for NA and PDA media), and whether this compound is the responsible of the growth of *A. thaliana* seedlings [36] remains to be elucidated.

In summary, results here reported confirmed that *Pseudomonas* sp. PICF6 and *P. simiae* PICF7 only antagonized *Verticillium* spp. suggesting that the mechanisms involved in in vitro antagonism of strains PICF6 and PICF7 are specific to a certain degree, or at least more effective, against representatives of this genus. Moreover, the characterization of the volatilomes of strains PICF6 and PICF7 allowed the identification of several compounds with known antimicrobial activity against different phytopathogens. However, the TCVA experiments indicated that none of the bacterial strains were able to inhibit the growth of *V. dahliae* by the action of volatiles, at least under our experimental conditions. Nevertheless, it must be stressed that the TCVA shows the effect of the total volatilome of bacterial strains and does not dissect the effect of individual compounds. From an agro-biotechnological point of view, and considering the antecedents available in the literature, the potential inhibitory effect against *Verticillium* spp. of specific compounds detected in the volatilomes of these beneficial rhizobacteria deserves further in-depth analysis.

## 4. Materials and Methods

### 4.1. Microorganisms and Growth Conditions

Olive root endophytic bacteria and fungal pathogens used in this study, including main features and references/source, are compiled in Table 4. Starting cultures of *Pseudomonas* sp. PICF6, *P. simiae* PICF7 and *P. polymyxa* PIC73 (Table 4), which belong to the culture collection of the Laboratory of Plant-Microorganism Interactions, Crop Protection Department, Institute for Sustainable Agriculture (IAS, Córdoba, Spain), were grown as described by Montes-Osuna et al. [35]. In all cases, inocula were spectrophotometrically (A600 nm) adjusted at 1·10^8^ cfu/mL. Fungi were previously grown at 25 °C in the dark in PDA medium (Oxoid, Basingstoke, UK).

### 4.2. In Vitro Antagonism Assays 

*Pseudomonas* sp. PICF6 and *P. simiae* PICF7 were tested against several relevant soil-borne fungal pathogens (Table 4). In addition, *P. polymyxa* PIC73 (Table 4) was included in the assays as reference due to its known broad-spectrum antagonistic activity against different plant pathogens [24]. Mycelial plugs (3-mm diameter) of each phytopathogen were obtained from 7-day-old colonies grown on PDA and placed in the center of Petri dishes (9 cm of diameter) with PDA or NA (Oxoid, Basingstoke, UK) media. Subsequently, four equidistant (2.5 cm from the center of the plate) 10 μL-drops of overnight cultures of each tested bacteria were inoculated around each pathogen. Additionally, Petri dishes inoculated only with mycelial plugs of each phytopathogen were used as controls. Plates were incubated at 25 °C until the pathogen covered the distance between microorganisms in the control plates (approximately 4 days for *R. solani* and *S. sclerotiorum*, 6 days for *F. oxysporum* f. sp. *lycopersici* Fol 007 and 14 days for *V. longisporum* ELV25 and *V. dahliae* V937I). The percentage of pathogen growth inhibition (relative inhibition index, PI) was calculated according to Gómez-Lama Cabanás et al. [25]. Experiments were performed twice with three biological replicates per each interaction and used medium.

### 4.3. Identification of the Volatilomes of Strains PICF6 and PICF7 

**Figure 1:**
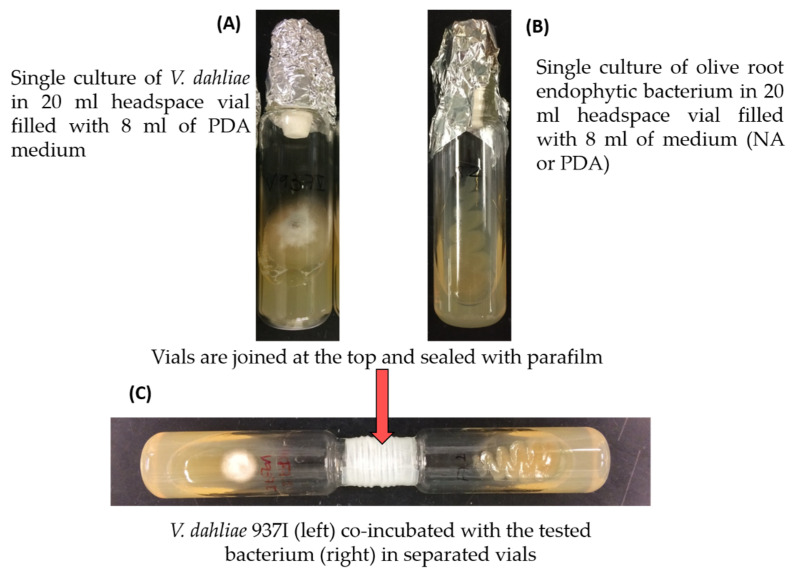
Culture headspaces sampling setup to characterize the volatile organic compounds of antagonistic olive root endophytes and *Verticillium dahliae* V937I. (**A**) *Verticillium dahliae* V937I, (**B**) bacteria and (**C**) bacteria in the presence of *V. dahliae*.

The volatilomes of the olive root endophytes *Pseudomonas* sp. PICF6 and *P. simiae* PICF7 were identified by HS-SPME GC-MS. Moreover, VOCs produced by these bacterial strains in the presence of *V. dahliae* V937I (co-incubated with the fungus in separated vials) were also determined to assess potential differences due to the presence of the target pathogen. In the latter case, and in order to discard possible compounds produced by *V. dahliae* V937I in the dual cultures, VOCs exclusively emitted by this fungal isolate were examined separately and used as control. Mycelial plugs (3 mm diameter) of *V. dahliae* V937I were placed in headspace vials (20 mL; 75.5 × 22.5 mm; Chromtech, Idstein, Germany) previously filled with 8 mL of PDA medium (Figure 1A). Bacterial isolates were streaked out in vials containing NA medium in parallel lines to assure equal distribution (Figure 1B). Vials with the BCA were connected to vials with the pathogen by the top of the container and sealed in this area with parafilm (Figure 1C). Three replicated vials were used for each BCA/pathogen combination, BCA or pathogen sample. In order to detect compounds that exclusively originated from the culturing media, vials filled only with PDA or NA were analyzed under the same conditions and used as controls. Vials were incubated at 25 °C for 5 days. Separation and detection of VOCs were performed on a system combining a GC 7890A with a quadrupol MS 5975C (Agilent Technologies GmbH, Waldbronn, Germany) as described by Mülner et al. [42]. For identification of microbial VOCs, the NIST MS Search 2.2 included in the Software-Package of the NIST 2014 database was used. Further verification was done by calculating the Kovats index (KI) and comparing it to database entries in the online database Chemspider (http://www.chemspider.com/, accessed on 17 February 2021).

### 4.4. Effect of Bacterial Volatiles on Mycelial Growth of Verticillium dahliae V937I

**Figure 2:**
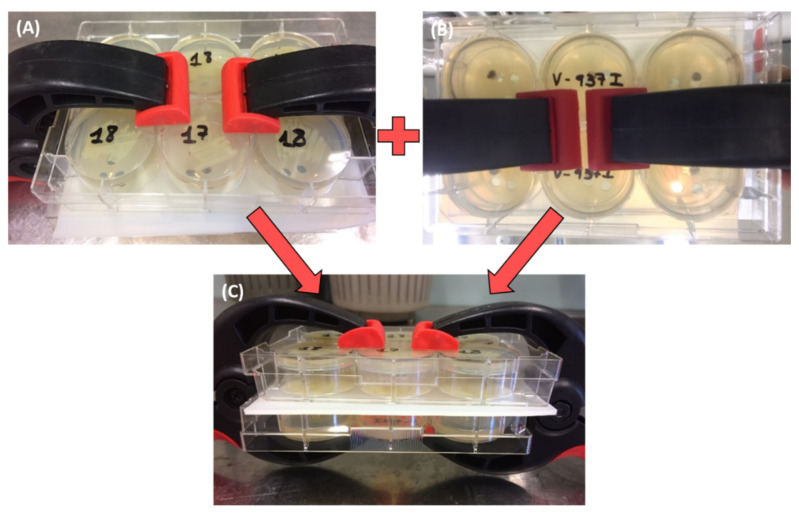
Evaluation of the ability of olive root bacterial endophytes to inhibit the mycelial growth of *Verticillium dahliae* V937I using the Two Clamp VOCs Assay (TCVA). Panel (**A**) shows the bacterial cultures placed on the top. Panel (**B**) shows the mycelial plugs of *V. dahliae* placed on the bottom. Panel (**C**) shows both plates (fungus and bacteria) connected by the perforated silicone foil and fixed with clamps.

The ability of *Pseudomonas* sp. PICF6 and *P. simiae* PICF7 to inhibit the mycelial growth of *V. dahliae* V937I was examined by using the Two Clamp VOCs Assay (TCVA), according to Cernava et al. [74]. Petri dish bottoms of a 6-well plate (Greiner Bio-One, Frickenhausen, Germany) were filled with 4 mL of either PDA or NA per well. Mycelial plugs (3 mm diameter) obtained from 7-day-old colonies grown on PDA were placed in the center of the wells of a 6-well plate containing PDA medium (Figure 2A). Subsequently, each bacterial sample was streaked out on the same position of a 6-well plate placed opposite to the plate with the pathogen (Figure 2B). Under this setup, the bacteria were tested on two different media, PDA and NA. A silicone perforated foil was placed between both 6-well plates to facilitate their fixing in combination with the usual clamps as shown in Figure 2C. As a control, wells containing *V. dahliae* were connected to wells filled only with PDA or NA (without any bacteria). Plates were incubated at 25 °C for 6 days. Subsequently, the largest and smallest diameters of the *V. dahliae* colonies were measured. Six replicates per each *Verticillium*-bacteria and *Verticillium*-medium (control) combination were performed.

### 4.5. Data Analysis

Analysis of variance (ANOVA) was performed to determine statistical differences using the ANOVA module of Statistix 10 program (NH Analytical Software, Roseville, MN, USA). Data were tested for normality, homogeneity of variances and subjected to whiskers and graphic boxes in order to detect the outlier, which proved their suitability for the statistical analysis in all experiments. When analysis of variance showed significant differences among treatments, means were compared according to Tukey honestly-significant-difference (HSD) test at *p* = 0.05.

## Figures and Tables

**Table 1:** Percentage of growth inhibition exerted over different fungal pathogens by *Pseudomonas* sp. PICF6 and *Pseudomonas simiae* PICF7.

Pathogens	*Ss*		*Rs*		*Fol* 007		ELV25		V937I	
Media	PDA	NA	PDA	NA	PDA	NA	PDA	NA	PDA	NA
**Bacterial strains**										
***Paenibacillus polymyxa***										
PIC73	72.62 **a**	68.44 **a**	59.23 **a**	51.15 **a**	52.94 **a**	-	66.89 **a**	56.25 **a**	Nd ^1^	Nd ^1^
***Pseudomonas***										
PICF6	-	-	-	-	-	-	33.78 **b**	21.88 **b**	40.96 **a**	29.72 **a**
PICF7	-	-	-	-	-	-	31.08 **b**	20.00 **b**	41.49 **a**	21.70 **a**

*Ss*, *Sclerotinia sclerotiorum*; *Rs*, *Rhizoctonia solani*; *Fol* 007, *Fusarium oxysporum f.* sp. *lycopersici Fol* 007; ELV25, *Verticillium longisporum* ELV25; V937I, *Verticillium dahliae* V937I; PIC73, *Paenibacillus polymyxa* PIC73; PICF6, *Pseudomonas* sp. PICF6; PICF7, *Pseudomonas simiae* PICF7. Values followed by different letters are significantly different (*p* ≤ 0.05) according to Tukey HSD Test in each column. At least three biological replicates for each dual confrontation and culturing medium were performed. PDA, Potato Dextrose Agar; NA, Nutrient Agar; -, no inhibition observed. This experiment was performed twice with similar results; Nd ^1^, not determined in this study. In vitro antagonism of this bacterium against *V. dahliae* isolates infecting olive has been previously demonstrated [24].

**Table 2:** Volatile organic compounds (VOCs) produced by the olive root endophytes *Pseudomonas* sp. PICF6 and *Pseudomonas simiae* PICF7, alone and during co-incubation with *Verticillium dahliae* V937I.

	VOCs in the Absence of *V. dahliae* V937I		VOCs in the Presence of *V. dahliae* V937I				
Predicted Compound (IUPAC)	PICF6	PICF7	PICF6	PICF7	Kovats Index	Reported Biological Functions	References
Methanethiol	x	x	x	x	401	n.a	
(Methylsulfanyl)methane	o	x	o	x	520	n.a	
S-Methyl ethanethioate	x	o	x	x	700	Antifungal activity	[53,54]
4-Methyl-2-pentanone	-	x	-	x	735	n.a	
(Methyldisulfanyl)methane	x	x	x	x	746	Antifungal activity, PGP	[47,55,56]
Bis(methylsulfanyl)methane	-	x	-	-	862	n.a	
(3E)-3-Nonene	x	x	x	x	889	n.a	
2,5-Dimethylpyrazine	x	x	o	-	917	Antifungal activity	[57]
Dimethyltrisulfane	o	x	x	x	970	Antifungal activity	[47]
1-Decene	x	x	x	x	989	Antifungal activity, PGP	[58,59]
1,10-Undecadiene	x	x	x	x	1081	n.a	
1-Undecene	x	x	x	x	1091	Antifungal activity	[60]
4-Methyl-2,6-bis(2-methyl-2-propanyl)phenol	-	-	x	x	1513	Antifungal activity	[61]
3,7-Dimethyl-1-octene	-	-	x	x	963	n.a	
Tridecane	o	x	x	-	1300	PGP	[62]
(3Z)-3-Dodecene	x	o	o	-	1185	n.a	
2-Decyloxirane	-	x	-	-	1307	Antifungal activity	[51]
2,6,11-Trimethyldodecane	o	-	o	-	1275	n.a	
Methyl thiocyanate	x	-	-	-	702	Antifungal activity	[54]
1-Tridecyne	-	-	o	-	1297	n.a	
2-Undecanone	-	-	x	-	1294	Antifungal activity	[47]
2-Undecanol	-	-	x	-	1308	Antifungal activity, nematicidal activity	[63,64]
2-Nonanol	-	-	o	-	1101	Nematicidal activity	[63]
10-Methyl-1-undecene	-	-	x	-	1157	n.a	

Compound names according to International Union of Pure and Applied Chemistry (IUPAC). The Kovats index (KI) of the compounds was calculated with an alkane series. n.a, information not available or unknown function; PGP, plant growth promotion; x, the compound was detected in the three technical replicas; o, the compound was detected in two out of the three technical replicas; -, the compound was never detected.

**Table 3:** Effect of volatile organic compounds (VOCs) produced by *Pseudomonas* sp. PICF6, *Pseudomonas simiae* PICF7 on the mycelial growth of *Verticillium dahliae* V937I evaluated using the Two Clamp VOCs Assay (TCVA).

	PDA	NA
Control	1.96 **a**	2.23 **a**
PICF6	1.73 **a**	1.91 **a**
PICF7	1.83 **a**	1.90 **a**

Data on mycelial growth (cm) are the means (*n* = 6), per each dual confrontation and media, between the largest and smallest diameters of the *V. dahliae* colony. Within each column, the same letter after mean values indicates no significant difference among treatments according to ANOVA test. PDA, Potato Dextrose Agar; NA, Nutrient Agar.

**Table 4:** Bacteria and fungi used in this study.

Microorganisms	Description	Reference/Source
**Bacterial strains**		
*Paenibacilluspolymyxa* PIC73	BCA	[24]
*Pseudomonas* sp. PICF6	BCA	[28,39]
*Pseudomonas simiae* PICF7	BCA/PGPR	[33,35]
**Fungal pathogens**		
*Fusarium oxysporum* f. sp. *lycopersici* Fol 007	Isolated from tomato roots (*Solanum lycopersicum*)	Graz University of Technology
*Rhizoctonia solani* Kühn	Isolated from potato tubers (*Solanum tuberosum*)	Graz University of Technology
*Sclerotinia sclerotiorum* (Lib.) de Bary 1884	Isolated from a bait system with sclerotia in potato tubers	Graz University of Technology
*Verticillium dahliae* V937I	Representative of the defoliating pathotype, originating from a diseased olive tree	[72]
*Verticillium longisporum* ELV25	Isolated from oilseed rape (*Brassica napus* L. ssp. *oleifera*) (Karin Zeise, Rostock)	[73]

BCA, biological control agent; PGPR, plant growth-promoting rhizobacteria. Fungal pathogens labelled with “Graz University of Technology” are part of the culture collection of the Institute of Environmental Biotechnolgy.

## Data Availability

All data required to reproduce the results presented in this study can be found in the article.

## Supplementary Materials

The following are available online at https://www.mdpi.com/article/10.3390/plants11030318/s1, Figure S1. In vitro antagonistic activity of *Pseudomonas* sp. PICF6 and *Pseudomonas simiae* PICF7 against *Verticillium longisporum* ELV25, Figure S2. Representative gas chromatography spectra of *Pseudomonas* sp. PICF6 and *Pseudomonas simiae* PICF7 (alone and in the presence of *Verticillium dahliae* V937I), *V. dahliae* 937I, PDA and NA media.
