# Supplementary material for: Identification of Volatile Organic Compounds Emitted by Two Beneficial Endophytic Pseudomonas Strains from Olive Roots
Source: Plants (Basel). 2022 Jan 25;11(3):318. doi: 10.3390/plants11030318 (PMC8840531; doi:10.3390/plants11030318)
Supplement: Supplementary file 1 [file plants-11-00318-s001.zip › plants-1542404-supplementary.pdf]

Supplementary material

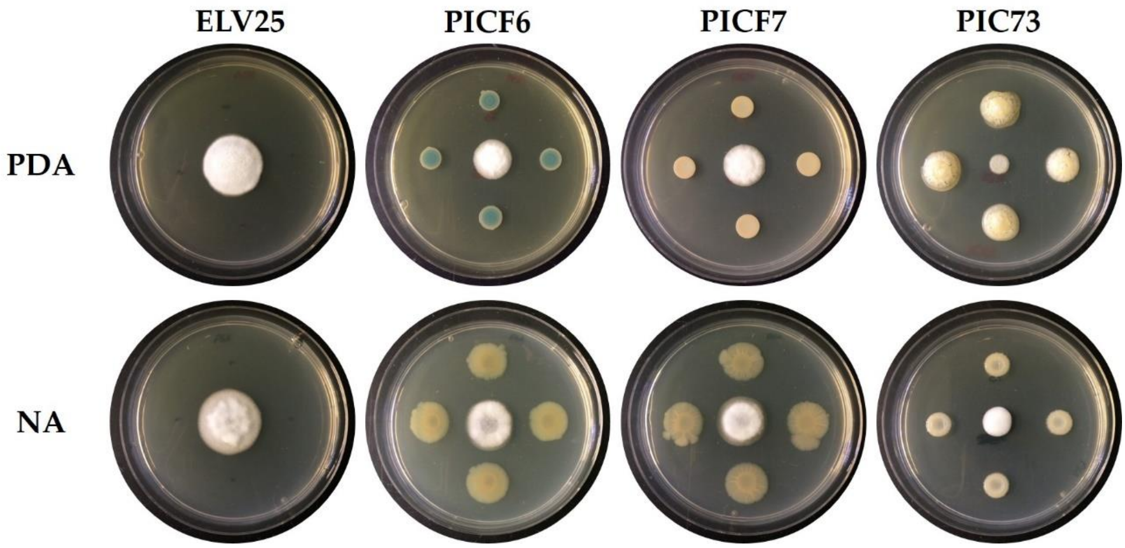

Figure S1. *In vitro* antagonistic activity of *Pseudomonas* sp. PICF6 and *Pseudomonas simiae* PICF7 against *Verticillium longisporum* ELV25. *Paenibacillus polymyxa* PIC73 was included in the assays as reference due to its known broad-spectrum antagonistic activity against different plant pathogens. Pictures were taken after 14 days of incubation at 25 °C. ELV25, *V. longisporum* ELV25; PICF6, *Pseudomonas* sp. PICF6; PICF7, *P. simiae* PICF7; PIC73, *P. polymyxa* PIC73; PDA, Potato Dextrose Agar; and NA, Nutrient Agar. Images correspond to representative plates from two independent experiments.

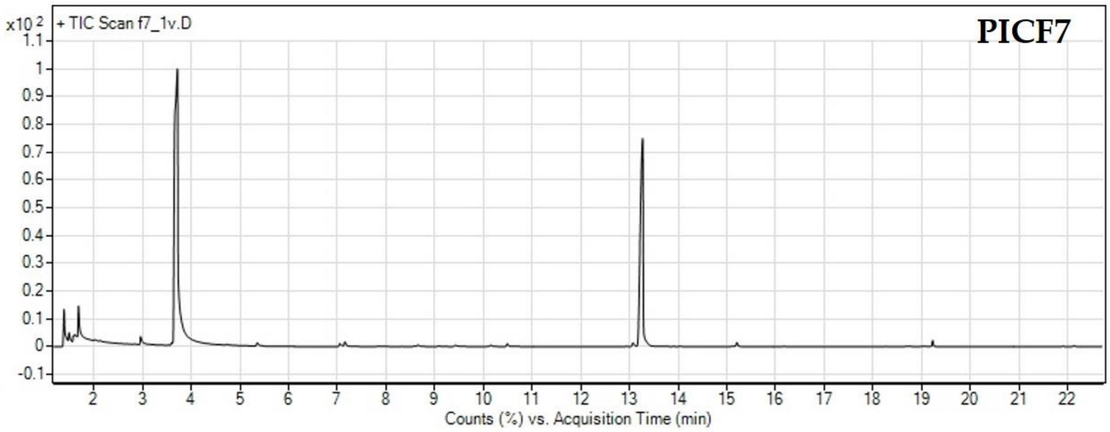
x10 2
+ TIC Scan f7_1v.D
PICF7
Counts (%) vs. Acquisition Time (min)

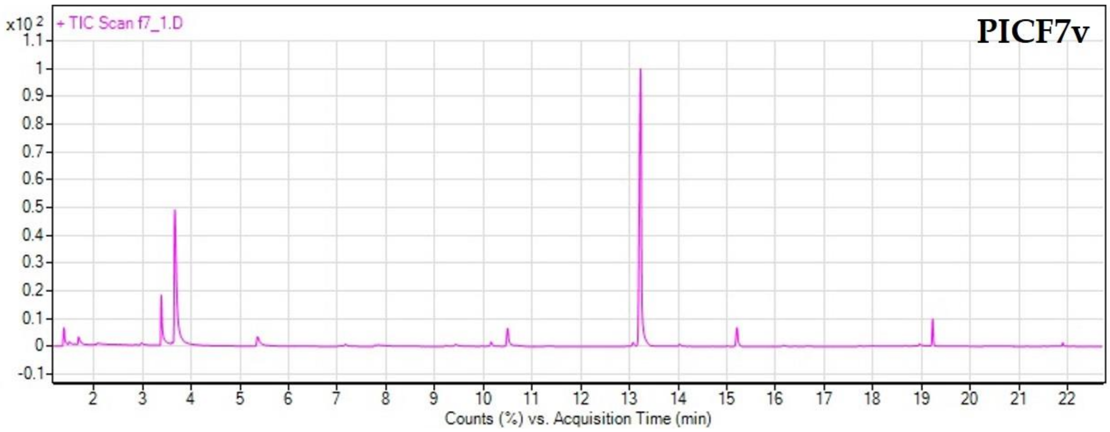
x10 2
+ TIC Scan f7_1.D
PICF7v
Counts (%) vs. Acquisition Time (min)

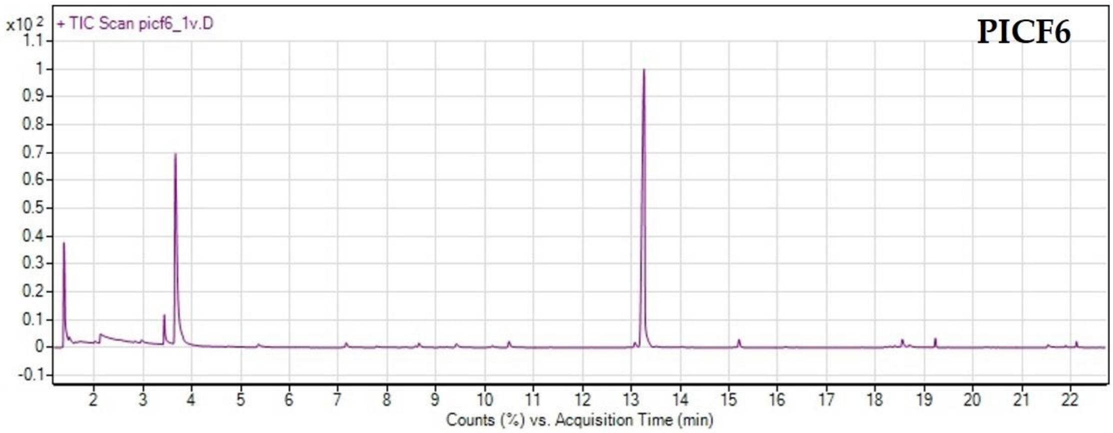
x10 2
+ TIC Scan picf6_1v.D
PICF6
Counts (%) vs. Acquisition Time (min)

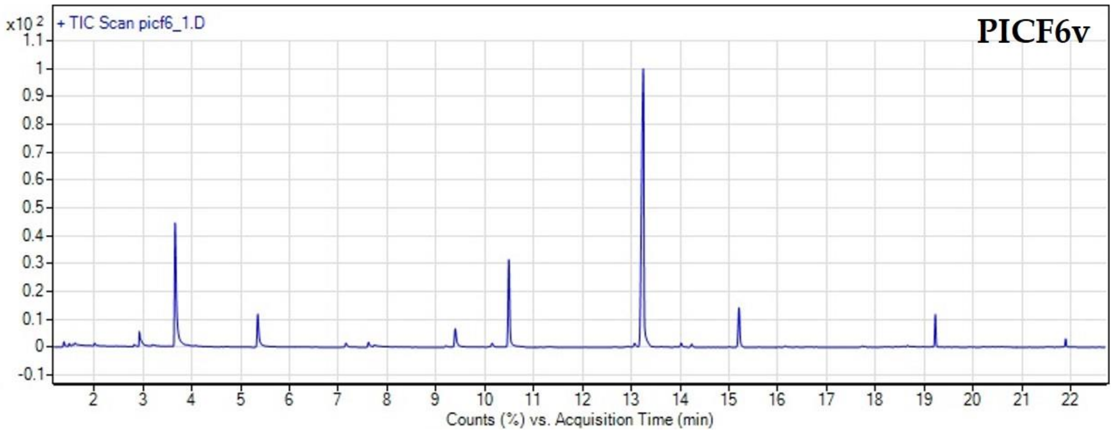
x10 2
+ TIC Scan picf6_1.D
PICF6v
Counts (%) vs. Acquisition Time (min)

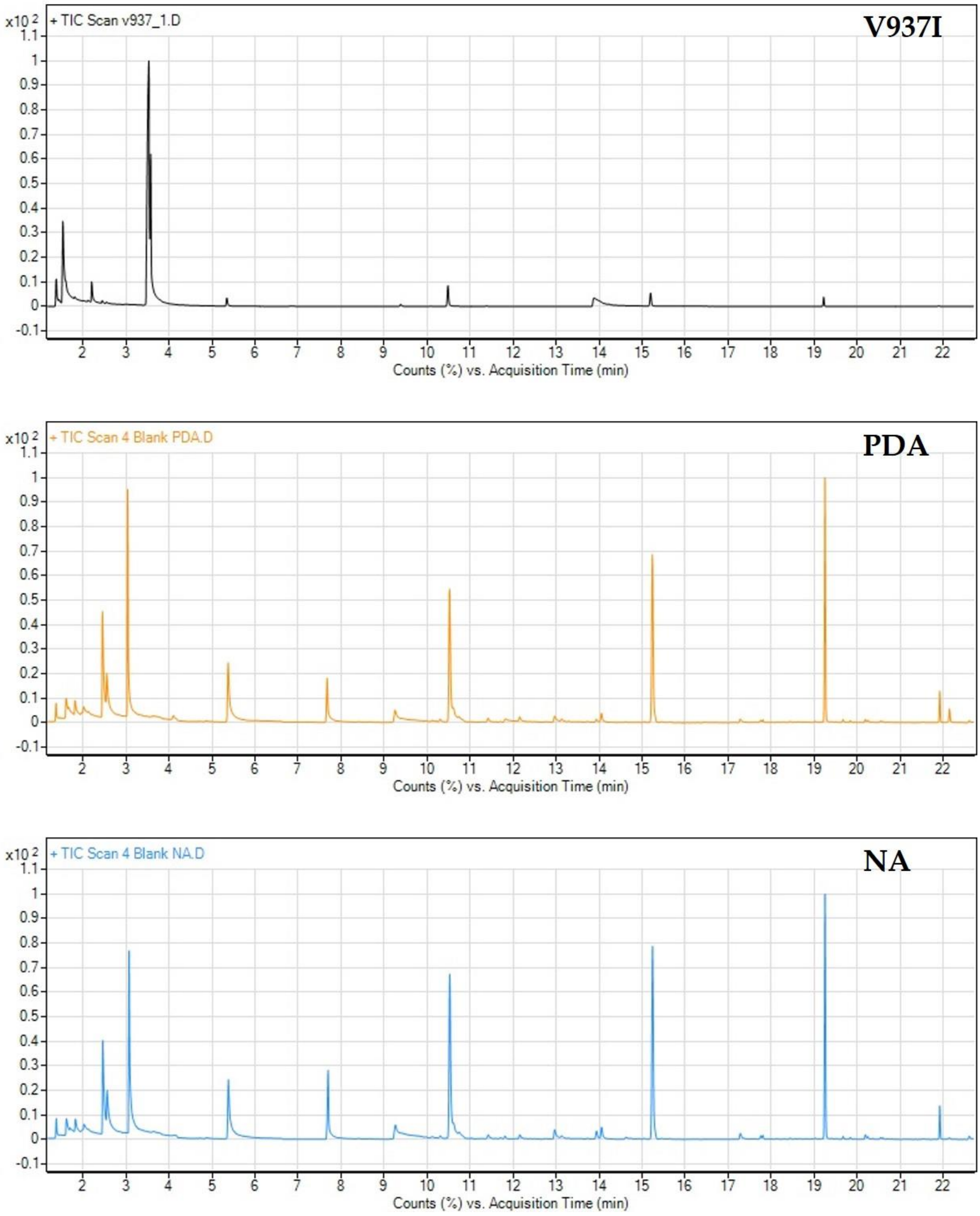

Figure S2. Representative gas chromatography spectra of *Pseudomonas* sp. PICF6 and *Pseudomonas simiae* PICF7 (alone and in the presence of *Verticillium dahliae* V937I), *V. dahliae* 937I, PDA and NA media. PICF6, *Pseudomonas* sp. PICF6; PICF6v, *Pseudomonas* sp. PICF6 in the presence of *V. dahliae* V937I; PICF7, *P. simiae* PICF7; PICF7v, *P. simiae* PICF7 in the presence of *V. dahliae* V937I; V937I, *V. dahliae* V937I; PDA, Potato Dextrose Agar; NA, Nutrient Agar.
